# Local injury and systemic infection in infants alter later nociception and pain affect during early life and adulthood

**DOI:** 10.1016/j.bbih.2020.100175

**Published:** 2020-11-10

**Authors:** Carly I. Gomes, Gordon A. Barr

**Affiliations:** a Department of Neonatology, Children’s Hospital of Philadelphia, Philadelphia, PA, USA; b Department of Anesthesiology and Critical Care Medicine, Children’s Hospital of Philadelphia and the Perelman School of Medicine at the University of Pennsylvania, 3615 Civic Center Boulevard, Philadelphia, PA, 19104, USA; c Department of Psychology, University of Pennsylvania, 425 S. University Avenue, Stephen A. Levin Building, Philadelphia, PA, USA

**Keywords:** Carrageenan, E-Coli, Ontogeny, Sensitive period, Formalin test, Conditioned place aversion

## Abstract

Newborns in intensive care are regularly exposed to minor painful procedures at developmental time points when noxious stimulation would be normally absent. Pain from these interventions is inconsistently treated and often exists concurrently with systemic infection, a common comorbidity of prematurity. Our understanding of the independent and combined effects of early painful experiences and infection on pain response is incomplete. The main goals of this research therefore were to understand how pain and infection experienced early in life influence future nociceptive and affective responses to painful stimuli. Rat pups were infected with E-coli on postnatal day 2 (PN2) and had left hind paw injury with carrageenan on PN3. Standard thermal tests for acute pain, formalin tests for inflammatory pain, and conditioned place aversion testing were performed at different ages to assess the nociceptive and affective components of the pain response. Early E-coli infection and early inflammatory injury with carrageenan both independently increased pain scores following hind paw reinjury with formalin on PN8, with effects persisting into adulthood in the carrageenan exposed group. When experienced concurrently, early E-coli infection and carrageenan exposure also increased conditioned aversion to pain in adults. Effect of sex was significant only in formalin testing, with males showing higher pain scores in infancy and females showing higher pain scores as adults. These findings demonstrate that infection experienced early in life can alter both the nociceptive and affective components of the pain response and that there is a cumulative effect of local and systemic pro-inflammatory processes on the aversive component of pain.

## 1 Introduction

Painful procedural interventions are a necessary aspect of neonatal intensive care, yet our knowledge of the effects of these interventions remains limited. Multiple epidemiological studies worldwide have shown that infants in NICU settings experience an average of 12 painful procedures each day, with the majority occurring in infants <30 weeks gestational age (Carbajal et al., 2008). These painful interventions occur during periods of rapid brain growth (Andescavage et al., 2017) at developmental time points where noxious stimulation is normally absent or limited (Ren et al., 2004a; Ruda et al., 2000). Moreover these painful exposures are often experienced concurrently with other comorbidities of prematurity including systemic infection (Joseph et al., 1998; Schrag and Stoll, 2006; Stoll et al., 2011). Although analgesic therapies are routinely provided for neonates undergoing major surgical procedures, they are not consistently provided for routine, minor painful procedures (Carbajal et al., 2008; Committee on fetus and newborn and Section on anesthesiology and pain medicine, 2016). As a result, these untreated early painful exposures produce acute hyperalgesia (Taddio et al., 2002, 2009) and short-term physiologic disturbances including vital sign changes, altered cerebral perfusion, and elevated salivary cortisol levels (Anand, 1998; Bouza, 2009). In the long-term these exposures are associated with anatomical changes in the brain, cognitive problems, behavioral issues, and altered nociceptive responses to pain (Andrews and Fitzgerald, 1994; Duerden et al., 2018; Grunau et al., 2006; Ranger et al., 2013; Taddio et al., 1995, 1997; Vinall et al., 2014).

The International Association for the Study of Pain defines pain as, “an unpleasant sensory and emotional experience associated with actual or potential tissue damage or described in terms of such damage.” (Loeser and Treede, 2008). A complete understanding of the newborn pain experience, therefore, requires study of both the nociceptive and affective components of pain. Newborns have the necessary neuroanatomical systems in place to detect a painful stimulus (Andrews and Fitzgerald, 1994; Bouza, 2009; Goksan et al., 2015), but the specifics of exactly how and where they process pain remains a mystery. Studies of nociception in animals have identified a “pain-sensitive” window in brain development during which exposure to noxious stimulation leads to long-lasting changes in pain response (Anand et al., 1999; Goksan et al., 2015; Lidow et al., 2001; Ruda et al., 2000) and anatomy (Anand et al., 1999; Andrews and Fitzgerald, 1994; Ruda et al., 2000). Animals exposed to painful inflammatory injury during this critical period have either minimally increased (hypoalgesia) or unchanged pain thresholds at baseline with increased frequency of nociceptive behaviors and increased nociceptive sensitivity (hyperalgesia) in the setting of subsequent inflammatory injury as adults (Lidow et al., 2001; Ruda et al., 2000; Wang et al., 2004). Anatomical changes observed include enlarged spinal cord receptive fields and associated enhancement in somatosensory activation following re-injury (Anand et al., 1999; Andrews and Fitzgerald, 1994; Ruda et al., 2000). Alterations in the neural axis after early painful injury have also been reported in humans with very premature infants showing white matter injury and decreased connectivity between cortical regions and the thalamus as early as 33 weeks gestational age and changes, including thinning of the cerebral cortex (Ranger et al., 2013), lasting until at least 8 years of age (Duerden et al., 2018; Vinall et al., 2014). Although considerable progress has been made in characterizing the nociceptive response to noxious stimulation in infancy, studies describing the affective response to pain remain limited.

Prematurity is associated with many comorbidities that adversely affect development and increase the need for invasive procedures, making establishment of causal relationships between early painful experiences and long-term behavioral effects challenging. Systemic E-coli infection is one common complication of prematurity that can lead to critical illness and increased need for painful interventions (Joseph et al., 1998; Schrag and Stoll, 2006; Stoll et al., 2011). A number of studies have shown that early infection (almost exclusively immune system activation with LPS) increases pain sensitivity in adults [(Campbell et al., 2015; Tadros et al., 2018; Wang et al., 2011; Yan and Kentner, 2017; Zouikr et al., 2014a, 2014b, 2015); reviewed in (Barr and Hunter, 2014; Karshikoff et al., 2019)]. Infection in the first postnatal week in rats elevates plasma and brain cytokines and chemokines and has delayed effects on the inflammatory pain response (Hunter et al., 2015; Yan and Kentner, 2017; Zouikr et al., 2015) including enhancement of mechanical allodynia and activation of both spinal and supraspinal structures like the prefrontal cortex and periaqueductal gray of the midbrain [PAG (Hunter et al., 2015; Yan and Kentner, 2017; Zouikr et al., 2014a, 2014b)]. The long-term behavioral effects of early E-coli infection, including decreased social interaction and increased response to stress (Bilbo and Schwarz, 2012; Bilbo et al., 2008), and limited effects on anxiety-like and depression-like behaviors (Yan and Kentner, 2017) have also been well-characterized in animal models. However, few studies have looked beyond the effects of early infection on the nociceptive component of pain and there are no studies that we are aware of that examine its effects on pain affect, arguably the most important aspect of pain processing.

As newborns in intensive care settings are commonly and concurrently exposed to *both* early painful experiences and early infection, it is critical to understand the combined effects of these experiences on behavior and development as they may differ from the effects of each alone. The main goals of this research were to understand how infection and pain experienced early in life alter future nociceptive and affective responses to subsequent painful experiences. We hypothesized that exposure to painful stimulation or infection early in life would be associated with heightened nociceptive and affective responses to subsequent painful stimuli experienced throughout development. Specifically, we hypothesized that early systemic infection with E-coli (PN2) or early inflammatory tissue injury with carrageenan (PN3) would each *independently* produce few changes or a mild hypoalgesia in baseline thermal sensitivity, produce hyperalgesia in the inflammatory formalin test, and produce increased aversion in a conditioned place aversion test on PN8, PN15, or in adulthood. We further hypothesized that *concurrent* experience of inflammatory pain and systemic infection in early postnatal life would produce heightened hyperalgesia after a later inflammatory injury with formalin and heightened aversion to painful stimulation over and above that seen with either insult alone. Both male and female animals were used to test if hypothesized effects were sex dependent.

Although there is published research on the long-term effects of infant pain in both humans and animal models [(Anand, 1998; Andrews and Fitzgerald, 1994; Bouza, 2009; Duerden et al., 2018; Ranger et al., 2013; Ruda et al., 2000; Taddio et al., 1995, 1997, 2002, 2009; Vinall et al., 2014; Wang et al., 2004); for reviews see (Grunau et al., 2006; Lidow et al.)] and on effects of neonatal infection on adult pain [(Campbell et al., 2015; Tadros et al., 2018; Wang et al., 2011; Yan and Kentner, 2017; Zouikr et al., 2014a, 2014b, 2015); for review (Karshikoff et al., 2019)], we are unaware of any studies describing the combined effects of infant pain and infant infection on future pain responses, nor on the effects of early pain, early infection, and their combination on pain affect. Increasing our knowledge of the long-term effects of the entirety of experiences that ill newborns undergo is a necessary first step in optimizing pain management and reducing enduring adverse effects in this vulnerable population.

## 2 Methods

### 2.1 Ethical guidelines

All experiments complied with ARRIVE guidelines, were approved in advance by The Children’s Hospital of Philadelphia IACUC, and were in accordance with the NIH Guide for the Care and Use of Laboratory Animals (NIH Publications No. 80–23), and with guidelines from national and international professional societies for the ethical treatment of animals. There were defined humane endpoints for painful interventions in the IACUC protocols but no animals reached those endpoints prior to the experimental endpoints.

### 2.2 Animals

Long-Evans Hooded rat pups were bred in our animal facility from our existing colony (Envigo), to avoid the stress of shipping pregnant dams (Kuwagata et al., 2009; Ogawa et al., 2007; Pritchett-Corning et al., 2013). Animals were housed with *ad libitum* food and water in rooms kept at 20–23 ​°C with a 12 ​h light-dark cycle with lights on at 0600 ​h. Pregnant dams were assessed twice daily for births of new litters at about 0900 ​h and 1700 ​h. The date of birth was designated as postnatal day 0 (PN0). Pups and dams were housed in standard cages (cage size: 51 ​× ​40cm) with free access to food, water, bedding, extra nest building materials, and a plastic enrichment tunnel. At PN21, pups were weaned and housed in same sex groups (4 per cage) until adulthood (2 per cage).

There were 25 litters utilized with 7–9 animals per litter (see Statistical section below for details about litter effects). Individual animals were distinguished from one another based on their naturally occurring color markings. Long-Evans hooded rats are born with hyperpigmented patches of skin with unique patterns that can be easily visualized throughout development and into adulthood. These patterns were used to identify individual animals over time thereby avoiding the need for painful tattooing, tail clipping, or ear punching (Koch, 2006).

### 2.3 E-coli culture

E-coli was used to induce systemic infection in pups (PN3). E-coli (ATCC 15746: Castellani and Chalmers) was rehydrated and cultured overnight in Tryptic Soy Broth (TSB) at 37 ​°C. Cultures were supplemented with 10% glycerol, aliquoted, and frozen at −80 ​°C. Aliquots were thawed and cultured overnight in fresh TSB the day prior to use. A microplate reader (Molecular Devices, San Jose, CA) was utilized to quantify bacteria based on previously obtained growth curves. Samples were centrifuged at 125 ​rpm for 2 ​min and pellets resuspended in fresh Phosphate Buffered Saline (PBS) to yield a concentration of 0.1 ​× ​10^6^ ​CFU E-coli per 0.1 ​mL. This protocol was adapted from the work of Bilbo and Schwarz (Bilbo et al., 2005a, 2008; Bilbo and Schwarz, 2012).

### 2.4 Experimental design

**Fig. 1:**
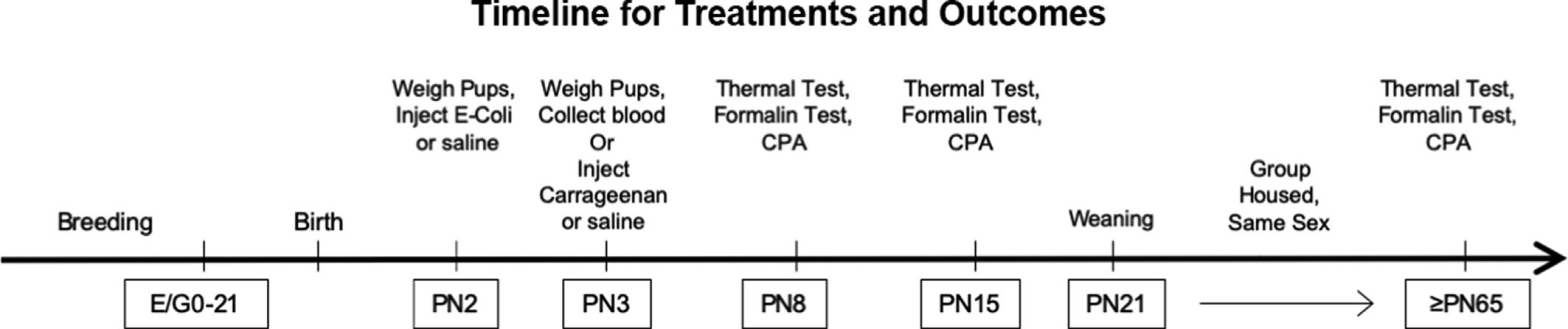
*Timeline for the experimental design.* Litters were checked twice daily for birth of new pups with the day of birth denoted PN0. Pups were inoculated on PN2 with an E−coli suspension or a saline control and weighed. On PN3 pups were reweighed and their left hind paws were injected with either carrageenan or saline. Trunk blood and brain stem tissue were also obtained from a random subset of pups on PN3 to assess for levels of ACTH/corticosterone in blood and interleukin-1Rβ in brain. Tests for thermal and formalin-induced nociception and conditioned place aversion to cues associated with the formalin test were performed at PN8, PN15, and in adults. (E/G: embryonic/gestational age; PN: postnatal day; CPA: conditioned place aversion test.

Fig. 1 is a schematic of the design and timeline of the experiments.

The neurological maturity of rat pups at the time of birth is approximately equal to 24 weeks of gestational age in humans (Semple et al., 2013; Workman et al., 2013). The age for infection (PN2) was therefore selected to mimic early-onset E-coli sepsis in premature human infants, a common cause of early immune system activation and clinical illness in babies born extremely preterm (Joseph et al., 1998; Schrag et al., 2016; Stoll et al., 2011). On PN2, pups were briefly separated from their mothers, weighed, and subcutaneously injected with either 0.1 ​mL of PBS or 0.1 ​× ​10^6^ ​CFU E-coli (ATCC 15746: Castellani and Chalmers) suspended in 0.1 ​mL of PBS (Bilbo et al., 2008). Though systemic E-coli infection in preterm infants can be severe and life threatening, the strain of E-coli used experimentally caused only self-limited infection in order to ensure survivability of pups to adulthood. Injections were performed on a uniformly heated surface (~30 ​°C) to prevent hypothermia and cold stress. Litters were assigned sequentially to receive either PBS (12 litters) or E-coli suspended in PBS (13 litters). All pups from a single litter received the same treatment with manipulations lasting <5 ​min total. Pups were then returned to their home cages simultaneously, thereby ensuring that littermates spent an equal amount of time away from their mother.

E-coli infection has been associated with slow weight gain and also markedly increases circulating cytokines and corticosterone short-term in rats (Bilbo et al., 2005a, 2005b; Wandall et al., 1997). Thus, to verify successful infection in our experiments, animals were weighed approximately 24 ​h after inoculation with E-coli or injection with saline and percent weight gain between groups was compared. Trunk blood was obtained at this time from a random subset of pups from multiple litters to assess for expected elevations in serum ACTH and corticosterone. Additionally, to confirm a CNS response to E-coli infection, brainstem tissue was obtained from single pups from two E-coli infected litters and two control litters and flash frozen under RNAse free conditions. Taqman primers for interleukin-1β were used and validated as previously described (Barr et al., 2016) and GADPH was used as the loading control primer. We specifically analyzed this particular brain region because immune markers and Fos protein levels have been shown to be elevated in brainstems of infected animals. Furthermore, activation of the brainstem in the setting of infection has been shown to produce apnea, a common clinical symptom seen in newborns with sepsis (Hofstetter et al., 2007).

On PN3 pups were re-identified by their markings, separated by sex, and randomly assigned to receive injection into the plantar surface of the left hind paw with either 0.25% carrageenan (1 ​μl/g) dissolved in sterile saline or sterile saline alone. Carrageenan is a commonly used inflammatory agent in pain research that causes localized erythema and swelling of the injected tissue (Laprairie and Murphy, 2007; Ren et al., 2004a) even when administered at low concentrations (Lidow et al., 2001). Observed effects are self-limited with resolution of symptoms by 24–48 ​h.

Pups were again kept on a uniformly heated surface when separated from their mother to prevent cold stress and returned to their home cages simultaneously. The time point for exposure to carrageenan was selected to fall within the pain-sensitive window (Ren et al., 2004a; Ruda et al., 2000) and to mimic the noxious stimulation and inflammatory injuries that extremely premature infants are exposed to in the NICU in early life (Carbajal et al., 2008).

### 2.5 Behavioral testing

In order to evaluate the effects of early noxious stimulation at different stages of development, behavioral testing was performed at one of three specified ages: PN8, PN15, or after PN65. The neurological maturity of rat pups at these ages, one week, 2 weeks, and 65 days, corresponds to that of humans at approximately full-term, mid-infancy, and adulthood, respectively (Semple et al., 2013; Workman et al., 2013). All behavioral testing was performed in designated rooms within the core animal facility at The Children’s Hospital of Philadelphia. Animals were randomly coded prior to testing and, only after behavioral testing was completed, were they re-identified using their unique skin/fur markings to determine from which experimental groups they came. Blind live scoring was done for each behavioral test for all ages, except for adult CPA tests for which only video was recorded. Simultaneous video was recorded for all formalin testing at all ages. When video scoring was available it was used for data analysis except in a few cases where technical problems precluded acquisition of video footage and data from live scoring was used instead. Two independent observers scored the videos and were blinded to all experimental conditions. All observers were trained by a single instructor who confirmed inter-observer reliability.

#### 2.5.1 Plantar thermal withdrawal test

We used a standard heat latency test paradigm (paw withdrawal latency to a thermal stimulus) to establish basal pain thresholds for each experimental condition at PN8, PN15, and in adulthood (Hargreaves apparatus: IITC Plantar Analgesia Meter (Hargreaves et al., 1988). This plantar thermal withdrawal test was used to assess the nociceptive response to acute pain. Rats were placed onto a uniformly heated glass surface maintained at 30 ​°C and allowed to habituate to their clear, plexiglass testing chambers for 30 ​min. A radiant heat lamp (24 ​V halogen lamp) focused through a convex lens to a 2 ​× ​4 mm spot at a fixed location at a fixed distance from the glass flooring focused the thermal stimulus to the plantar surface of each hind paw. The length of time from initiation of the thermal stimulus to complete withdrawal of each paw from the glass surface was recorded (latency). We used a 20 ​s cutoff to avoid tissue damage. The behaviors were scored live for all ages.

#### 2.5.2 Formalin test

Standard formalin testing was used to measure nociceptive sensitivity in response to inflammatory pain (Guy and Abbott, 1992). A dilute solution of formalin in PBS (1 ​μl/g of 1% formalin in pups, 50 ​μl of 2% formalin in adults) was subcutaneously injected into the plantar surface of the left hind paw. Animals were then immediately placed into testing chambers on a uniformly heated surface (30 ​°C) to maintain temperature stability. A neutral lemon odor (Pure Lemon Extract, McCormick’s) was paired with the formalin injury in anticipation of subsequent Conditioned Place Aversion (CPA) testing. The testing chamber for adults also had striped walls and a smooth plexiglass flooring in addition to the lemon odor. Pain behaviors were scored based on increasing severity (Guy and Abbott, 1992) using an adapted 5-point scale (Barr et al., 2003; Butkevich et al., 2006). The scoring scale was as follows: 0 ​= ​no noticeable change in pain behavior with no favoring of the injected paw; 1 ​= ​favoring or decreased weight bearing on the injected paw with the paw still in contact with the floor; 2 ​= ​lifting of the entire injected paw completely off the floor; 3 ​= ​lifting of the entire injected paw completely off the floor with vigorous swiping or shaking of the paw; 4 ​= ​licking of the injected paw. Behaviors were scan sampled at 1-minute intervals with a total observation time of 30 (adults) or 45 (pups) minutes after injection. Behavioral scores were averaged into 3-minute periods to reduce variability (Barr, 1998; Butkevich et al., 2006). A subset of control animals from each experimental condition were similarly handled and observed but received no formalin injection. Saline was not injected in these controls since that itself is a painful stimulus. All formalin testing was video recorded with behaviors blindly scored from video footage, except in a few cases where live scoring data was used because of technical problems with the video recordings. Animals were returned to their home cages immediately after testing was complete.

#### 2.5.3 Conditioned place aversion test (CPA)

Standard CPA testing was used to study the affective component of the pain response and to assess for learned aversions commonly associated with pain (Minami, 2009). Previous studies in our laboratory and by others have demonstrated that rats are capable of having learned preferences at PN3 and PN8 (Barr and Rossi, 1992; Sullivan et al., 2000) with learned aversions demonstrated at PN3 and PN15 (Barr et al., 1994; Sullivan et al., 2000). CPA testing protocols for pups and adults were adapted from previous work (Barr and Goodwin, 1997; Barr et al., 1985, 1994).

All infant experiments were done within a heated (30–32 ​°C) air-jacked, sound attenuated incubator (Jeio Tech, Model IB-05G) to prevent hypothermia and cold stress. The infant test chamber was a small plexiglass box with wire-mesh flooring and a chamber directly below the flooring where cotton balls could be placed. The chamber was divided into three zones: a smaller neutral zone in the center flanked by two larger outer zones. One lemon scented cotton ball was placed beneath the mesh flooring on one side of the chamber (CS+) and a second unscented cotton ball was placed beneath the mesh flooring on the opposite side of the chamber (CS-). Three hours after formalin injury, pups were placed on the neutral zone and their movements were observed in four 2-minute intervals for a total of 8 ​minutes. After each 2-minute interval, pups were re-placed into the neutral zone in alternating orientations to avoid position biases. The length of time spent within each zone was recorded.

The adult testing chamber was divided into three compartments separated by portholes. The outer compartments were of identical size and shape, whereas the central compartment was smaller. Of the larger compartments, one was identical to the chamber where the rats were exposed to formalin injury of the hind paw: striping of the walls, smooth plexiglass floor, with the lemon scent. The opposite chamber was of the same size and shape but with a distinct sensory environment: plain walls, wire mesh flooring, and no odor. Twenty-four hours after formalin injury, rats were placed into the central, neutral chamber, and allowed to explore freely either side. The length of time spent within each chamber was recorded.

Observed behaviors were scored live for testing done at PN8 and PN15 and from videos in adults.

### 2.6 Statistics

All data were analyzed by parametric statistics. In Table 1 we present the number of litters (L), the number of independent data points analyzed (N), and the total number of pups tested shown in parentheses. Data presented for each litter represent the mean of all pups in that litter exposed to identical experimental conditions. Thus the litter was the experimental unit of analysis; this reduced variability and increased power and is the accepted procedure for analysis of data from multiparous species (Abbey and Howard, 1973; Festing, 2006; Wainwright, 1998). A total of 25 litters was used in our final analysis. One litter was excluded from analysis after the development of skin infection in several pups. Affected animals in that litter were euthanized in accordance with ethical standards as described above.

**Table 1:** Statistical analysis of results of behavioral testing throughout development.

	PN8		PN15		ADULT	
**THERMAL TEST**	L=14, N=27 (30)		L=16, N= 30 (35)		L=12, N=23 (34)	
E-coli	F (1,23) ​= ​0.015	p ​= ​0.903	F (1,26) ​= ​0.168	p ​= ​0.685	F (1,19) ​= ​0.046	p ​= ​0.833
Carrageenan	F (1,23) ​= ​0.237	p ​= ​0.631	F (1,26) ​= ​0.011	p ​= ​0.919	F (1,19) ​= ​1.368	p ​= ​0.257
Carrageenan x E-coli	F (1,23) ​= ​0.213	p ​= ​0.649	F (1,26) ​= ​1.209	p ​= ​0.282	F (1,19) ​= ​0.050	p ​= ​0.825
**FORMALIN TEST**	L ​= ​16, N= ​31 (32)		L ​= ​17, N ​= ​34 (34)		L ​= ​13, N ​= ​32 (33)	
E-coli	***F(1,24) ​= ​8.718***	***p ​= ​0.007***	F (1, 26) ​= ​4.056	p ​= ​0.054	F (1,25) ​= ​0.578	p ​= ​0.454
Carrageenan	***F(1,24) ​= ​5.902***	***p ​= ​0.023***	F (1, 26) ​= ​0.010	p ​= ​0.921	***F (1,25) ​= ​4.791***	***p ​= ​0.038***
Sex	***F(1,24) ​= ​5.384***	***p ​= ​0.029***	F (1, 26) ​= ​0.002	p ​= ​0.962	***F (1,25) ​= ​28.370***	***p ​< ​0.001***
Carrageenan x E-coli	F (1,24) ​= ​0.421	p ​= ​0.523	F (1, 26) ​= ​0.222	p ​= ​0.642	F (1,25) ​= ​0.118	p ​= ​0.734
Sex difference x E-coli	***F(1,24) ​= ​5.090***	***p ​= ​0.033***	F (1, 26) ​= ​1.104	p ​= ​0.317	F (1,25) ​= ​0.170	p ​= ​0.684
Carrageenan x Sex	F (1,24) ​= ​1.366	p ​= ​0.254	F (1, 26) ​= ​0.110	p ​= ​0.743	F (1,25) ​= ​0.410	p ​= ​0.528
Carrageenan x Sex x E-coli	F (1,24) ​= ​0.094	p ​= ​0.761	F (1, 26) ​< ​0.047	p ​= ​0.830	F (1,25) ​= ​0.034	p ​= ​0.856
**CONDITIONED PLACE AVERSION TEST**	L ​= ​13, N ​= ​25 (30)		L ​= ​15, N ​= ​28 (30)		L ​= ​13, N ​= ​21 (33)	
E-Coli	F (1,21) ​= ​0.373	p ​= ​0.547	F (1,24) ​= ​2.370	p ​= ​0.136	F (1,17) ​= ​0.325	p ​= ​0.576
Carrageenan	F (1,21) ​= ​0.094	p ​= ​0.762	F (1,24) ​= ​0.239	p ​= ​0.629	***F (1,17) ​= ​5.631***	***p ​= ​0.030***
Carrageenan x E-coli	F (1,21) ​= ​0.017	p ​= ​0.898	F (1,24) ​= ​1.950	p ​= ​0.175	***F (1,17) ​= ​5.728***	***p ​= ​0.028***

Note: The table outlines N’s, F-values, df, and p-values for behavioral testing conducted at PN8, PN15, and adulthood for the three behavioral tests performed: plantar thermal withdrawal test, formalin test, and conditioned place aversion test. Variables used in statistical analysis are listed in the left hand column. Results are divided into columns by developmental time point with significant values highlighted in bold and italicized print. If multiple animals from the same litter were exposed to identical experimental conditions their results were averaged and used as a single data point. The numbers above the statistic entries represent: the number of litters per experimental condition (L); the number of data points used in the data analyses (N) followed by the total number of pups tested shown in parentheses. This latter number accounts for pups for which the data were averaged.

Independent t-tests were used to compare differences in ACTH, corticosterone, and weight between groups inoculated with E-coli vs. saline. For all behavioral tests, we included sex as a variable in the initial analyses (3-way ANOVA: sex, E-coli infection, and carrageenan inflammation as variables). There were male-female differences only in formalin testing so data for this behavioral test includes sex as a variable. No other analyses found sex differences and thus subsequent analyses and figures combined the male and female data (2-way ANOVA: E-coli infection and carrageenan inflammation as variables). Likewise, for the analysis of time (averaged into 3 ​min bins) for the formalin test, there were no bin ​× ​treatment interactions and all subsequent analyses collapsed the data over time. Holmes- Šidák posthoc tests were used in cases of significant interactions using GraphPad’s Prism, V 8.4.0 to 9.0 for the MacOS. A detailed summary of our results and statistical analysis is included in Table 1.

**Fig. 2:**
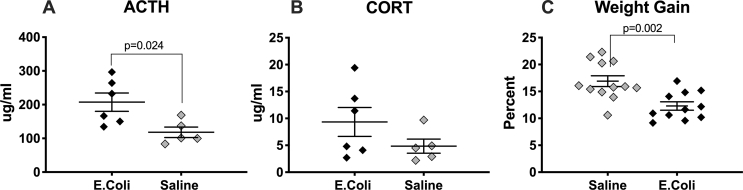
*Comparison of serum ACTH levels, serum corticosterone levels, and percent weight gain 24h after injection with E-coli suspension vs. saline control.* Pups were injected on PN2 with either PBS (saline) or E-coli suspended in PBS (E-coli). Trunk blood levels of ACTH and corticosterone were obtained 24 ​h after the E-coli injection. In panels **A** and **B**, the X-axis denotes groups of infected animals (E-coli) and controls (saline). The Y- axis shows μg/ml of ACTH or corticosterone, respectively. Each point represents an average of values obtained from animals within one litter of the same condition and error bars indicate ​± ​one SEM. ACTH levels were significantly higher in the group injected with E-coli suspended in PBS (t (9) ​= ​2.706, p ​= ​0.024), consistent with successful infection. Corticosterone levels were also higher in this group, although not significantly (t (9) ​= ​1.410, p ​= ​0.192). **C.** Pups were weighed daily on PN2 and PN3, immediately before and 24 ​h after injection with either PBS (saline) or E-coli suspended in PBS (E-coli). The X-axis denotes groups of infected animals (E-coli) and controls (saline). Percent weight gained for each is shown on the Y-axis. Percent weight gain was significantly lower in the litters injected with E-coli suspended in PBS (t (21) ​= ​3.547, p ​= ​0.002), consistent with successful infection.

**Fig. 3:**
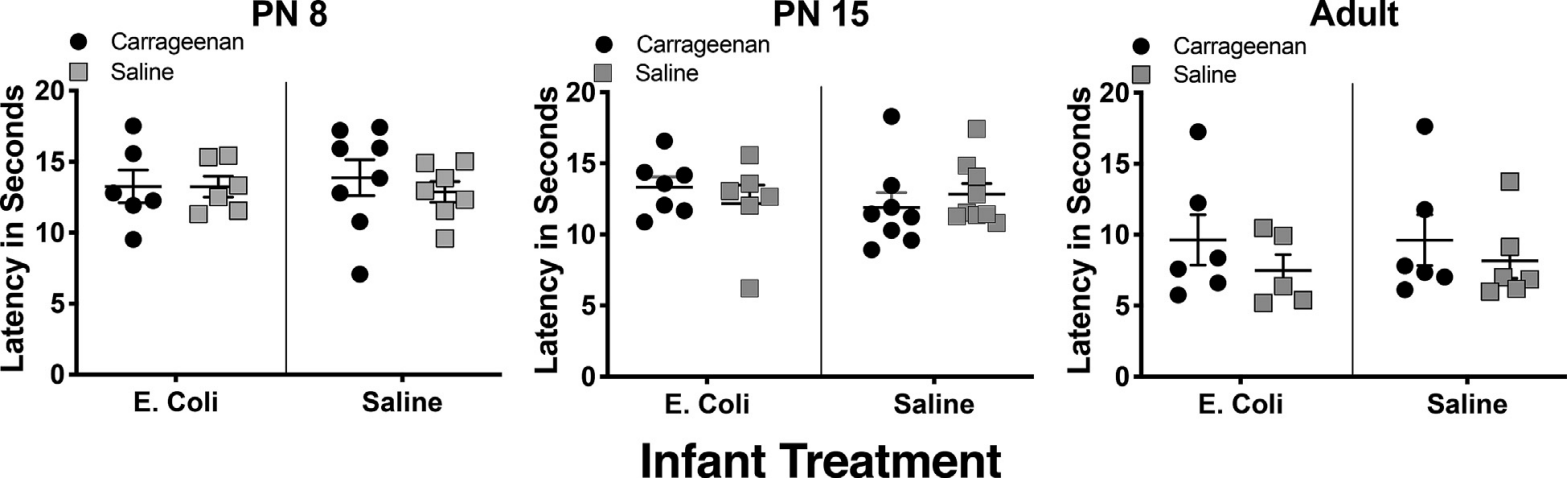
*Effects of early injury with carrageenan and early infection with E-coli on baseline heat latencies at various developmental time points.* Withdrawal latency to the thermal stimulus was measured in tenths of seconds for the two hind paws on PN8, PN15, or in adulthood. A 20 ​s cut-off was used to prevent tissue injury. The age when behavioral testing was performed is above each panel. The X-axis denotes groups of infected animals (E-coli) and controls (saline). Carrageenan treated animals are denoted by filled circles whereas the saline controls are denoted by the shaded squares. The latency to paw withdrawal in seconds is on the Y-axis. Error bars indicate ​± ​one SEM. Each point represents an average of values obtained from animals within one litter of the same experimental condition. Neither early hind paw injury with carrageenan nor early infection with E-coli had significant effects on heat latencies at any time point.

**Fig. 4:**
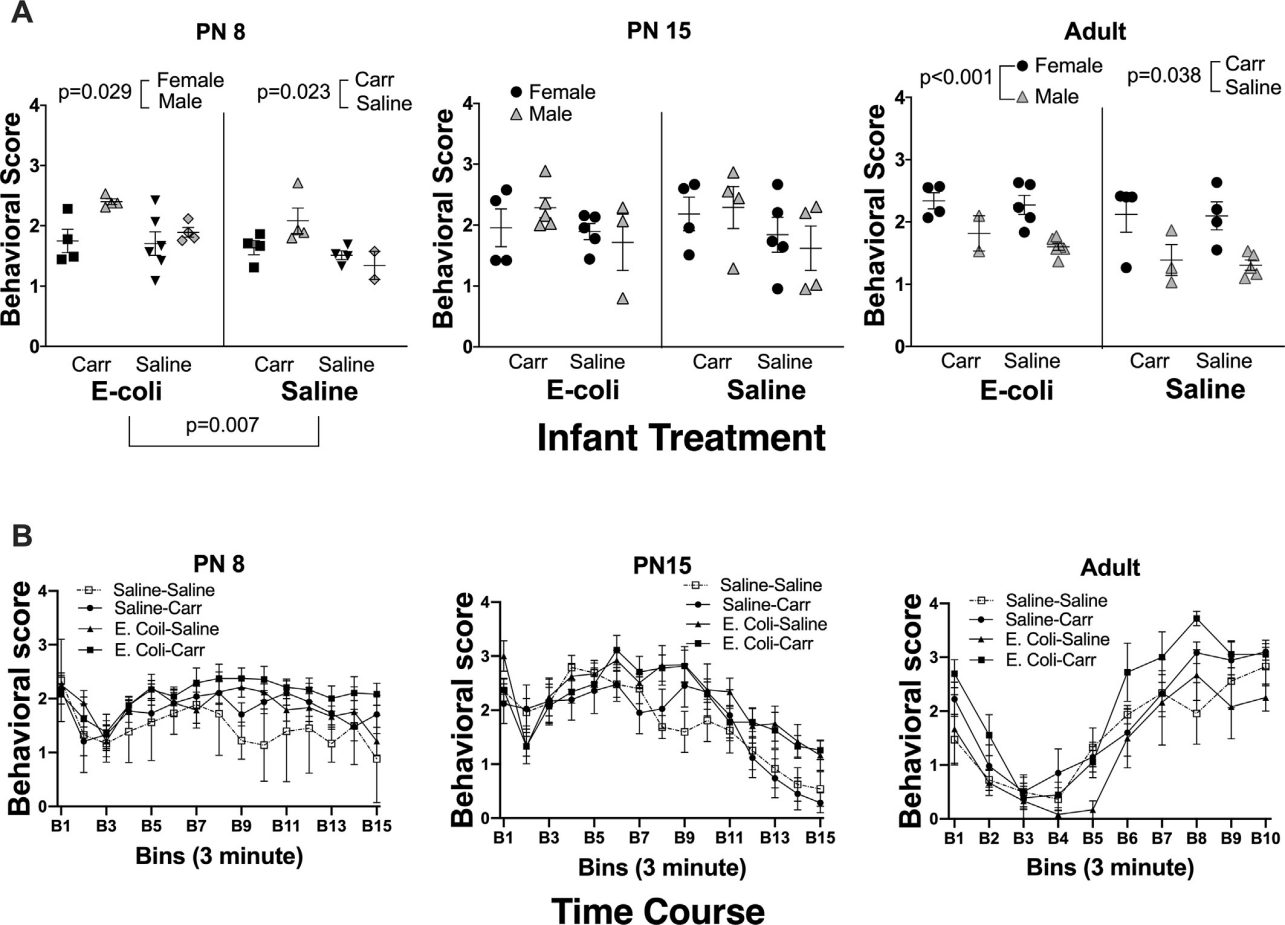
*Effects of early injury with carrageenan and early infection with E-coli on pain response following subsequent injury with formalin at various developmental time points.***A**. This panel of three ages shows average pain scores observed after formalin injury. The age when behavioral testing was performed is above each graph. The nociceptive response following hind paw injury with formalin was scored once per minute using a five point scale ranging from 0 to 4. Animals were observed for 30 (adults) or 45 (pups) minutes. Pain scores were averaged and shown on the Y-axis. The X-axis denotes the experimental groups subdividing animals based on their exposure to E-coli and/or carrageenan. Each point, females in filled circles and males in shaded triangles, represents an average of the individual pain scores obtained each minute during the full observation period. Error bars indicate ​± ​one SEM. If multiple animals from the same litter were exposed to the same condition, their results were averaged. Pain scores following reinjury with formalin on PN8 were significantly and independently increased by carrageenan exposure (F (1,24) ​= ​5.902, p ​= ​0.023), E-coli infection (F (1,24) ​= ​8.719, p ​< ​0.007), and in males vs. females (F (1,24) ​= ​5.398, p ​= ​0.029). Pain scores following injury with formalin in adulthood were also significantly increased by carrageenan exposure (F (1,25) ​= ​4.791, p ​= ​0.038) and in females (F (1, 25) ​= ​28.360, p ​< ​0.001). **B**. This panel shows a time course of the pain behaviors during the observation period. The age when behavioral testing was performed is above each panel. The X-axis denotes time, broken down into 3 ​min bins with initial formalin injury occurring at time 0. The nociceptive response is on the Y-axis.

For statistically significant effects, we calculated Cohen’s d’s to obtain two-group comparison effect sizes. These are presented in Table 2 and with the qualitative labels as described by Cohen (1988), and elaborated by Sawilowsky (2009). Estimation graphs of the effect size for each significant main effect and interaction are shown in Supplementary Fig. 1, Fig. 2, Fig. 3, Fig. 4. These were calculated in Prism as described: (https://www.graphpad.com/support/faq/creating-an-estimation-plot-of-the-results-of-an-unpaired-*t*-test/).

**Table 2:** Cohen Effect Size data.

Test and Age	Comparison	*t*-test	Cohen’s d	Interpretation
Weight Gain; PN2-PN3	E-coli vs Saline	t ​= ​3.574, df ​= ​21	1.503	Very large
		p ​< ​0.002		
ACTH; PN3	E-coli vs Saline	t ​= ​2.706, df ​= ​9	1.690	Very large
		p ​= ​0.024		
Formalin test; PN8	Carrageenan vs Saline	t ​= ​1.552, df ​= ​30	0.556	Medium
		p ​= ​0.131		
Formalin test; PN8	E-coli vs Saline	t ​= ​2.311, df ​= ​30	0.829	Large
		p ​= ​0.028		
Formalin test; PN8	Carrageenan-E-Coli-Male vs Saline-Saline-Male (Sex X E-coli)	t ​= ​6.218, df ​= ​4	4.276	Huge
		p ​= ​0.003ˆ		
Formalin test; PN8	Female vs Male	t ​= ​2.634 df ​= ​31	0.911	Large
		p ​= ​0.013		
Formalin test; Adult	Carrageenan vs Saline	t ​= ​1.579, df ​= ​32	0.564	Medium
		p ​= ​0.124		
Formalin test; Adult	Female vs Male	t ​= ​5.987, df ​= ​20	2.094	Huge
		p ​< ​0.001		
CPP; Adult	Carrageenan-E-coli vs Saline-E-coli (Carr X E-coli)	t ​= ​4.274, df ​= ​7	2.690	Huge
		p ​= ​0.004ˆˆ		
CPP; Adult	Carrageenan vs Saline	t ​= ​1.778, df ​= ​19	0.785	Medium
		p ​= ​0.091		

**Note:** The table shows the effect size for each significant main effect and two interaction effects. The experimental condition is in the first column; the specific comparison for that condition is in the second column. The third column contains the statistics for the subsequent *t*-test for the specific comparison. Cohen’s d calculations are in the 4th column and the interpretation of that effect shown in the rightmost column is from Cohen (Cohen, 1988) as elaborated by Sawilowsky (Sawilowsky, 2009).

Below are the probabilities from the posthoc Holm-Šidák tests for the interaction from the omnibus ANOVA.

ˆHolm-Sidak posthoc test: p ​= ​0.034.

ˆˆHolm-Sidak posthoc test: p ​= ​0.013.

## 3 Results

### 3.1 Infection

Animals injected with E-coli had elevated levels of serum ACTH (t (9) ​= ​2.706, p ​= ​0.024; Cohen’s d ​= ​1.690), significantly less weight gain than control animals (t (21) ​= ​3.574, p ​= ​0.002; Cohen’s d ​= ​1.503), and a two-fold increase in interleukin-1β (^ΔΔ^CT ​= ​2.02; data not shown) 24 ​h after inoculation. Corticosterone levels were also elevated, but not significantly so (t (9) ​= ​1.410, p ​= ​0.192; Fig. 2). As inflammatory markers are elevated following development of systemic infection in human infants and impaired weight gain is seen in animals with systemic infection, the increased levels of ACTH and interleukin 1β and slower weight gain observed in E-coli inoculated animals verified that these animals were successfully infected.

### 3.2 Plantar thermal withdrawal test

The initial 3-way ANOVAs showed no effect of sex at any age (all p values ​> ​0.063) and therefore males and females were combined and data analyzed by 2-way ANOVAs. There were no significant differences in thermal baseline pain thresholds in carrageenan exposed pups versus saline controls at any age; infection with E-coli did not alter these findings (all p values ​> ​0.257; Table 1; Fig. 3). When the paw injected with carrageenan was compared to the uninjected paw, there were also no differences (Supplemental Figure 5 and Supplemental Table 1). Thus, neither early injury with carrageenan nor infection with E-coli, or their combination, altered baseline thermal thresholds.

### 3.3 Formalin test

There were significant sex differences in the pain response during formalin testing at PN8 and in adulthood and sex was included as a variable in all subsequent analyses. Nociceptive scores following injury of the left hindpaw with formalin were altered by carrageenan, infection, and sex at different developmental time points (Table 1, Fig. 4). Infection with E-coli on PN2 independently increased nociceptive scores on PN8 (F (1,24) ​= ​8.718, p ​= ​0.007; Cohen’s d ​= ​0.829) with males showing higher nociceptive sensitivity (hyperalgesia) to formalin injury as compared to females (F (1,24) p ​= ​5.384, p ​= ​0.029; Cohen’s d ​= ​0.911). There was also an interaction of Sex with E-coli at this age (F (1,24) ​= ​5.090, p ​= ​0.033) with posthoc analysis showing that E-Coli/Carrageenan males differed from Saline/Saline males (p ​= ​0.034; Cohen’s d ​= ​4.276). Injury with 0.25% carrageenan on PN3 also significantly increased pain scores following reinjury with formalin on PN8 (F (1,24) ​= ​5.902, p ​= ​0.023; Cohen’s d ​= ​0.556), demonstrating an independent effect of early inflammatory injury on future pain response (Fig. 4A). There were no significant effects of any variables at PN15 (all p’s ​> ​0.050). In adults, there were no significant independent effects of E-coli on pain behaviors (F (1,25) ​= ​0.578, p ​= ​0.454). Nociceptive scores were higher in carrageenan treated rats (F (1,25) ​= ​4.791, p ​= ​0.038; Cohen’s d ​= ​0.564) and in females (F (1, 25) ​= ​28.370, p ​< ​0.001; Cohen’s d ​= ​2.094) with no other significant main effects or interactions noted. Thus, both early E-coli infection and early injury with carrageenan produced hyperalgesia to inflammatory pain at PN8 with carrageenan effects persisting to adulthood. There were also sex differences at both PN8 and adulthood, with infant males and adult females showing hyperalgesia to subsequent injury.

### 3.4 Conditioned place aversion test

**Fig. 5:**
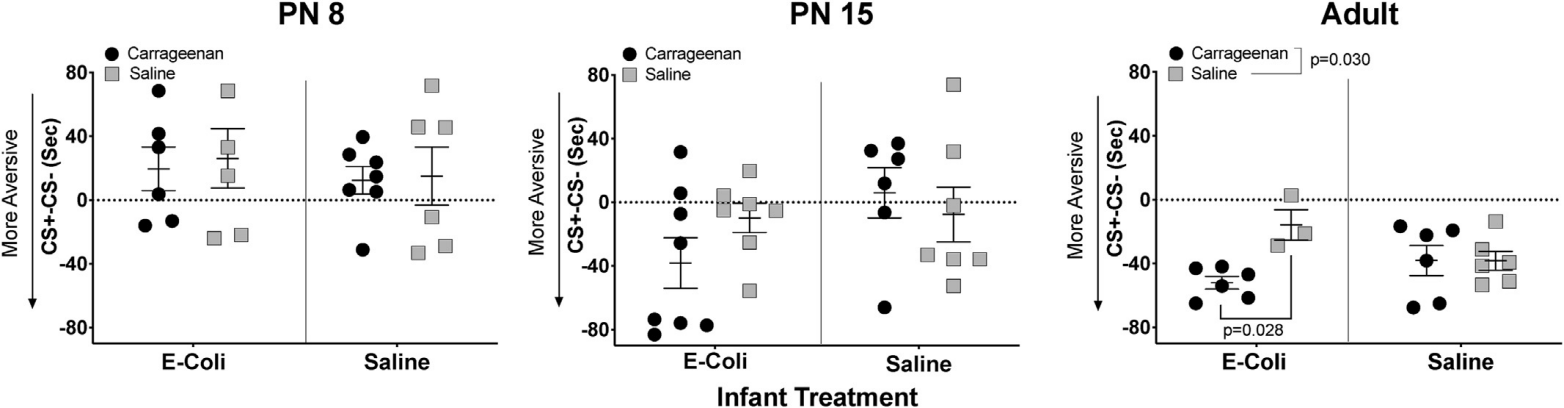
*Effects of early carrageenan injury and early infection on learned aversion to painful stimuli.* The age when behavioral testing was performed is above each panel. The Y axis is a measure of aversion and denotes the relative amount of time spent in the testing chamber paired with the painful stimulus. On this scale, positive numbers reflect more time spent within the testing chamber paired with the painful stimulus and indicate a relative preference for this chamber. Negative numbers reflect less time spent within the testing chamber paired with the painful stimulus and indicate a relative aversion to this chamber. The X-axis denotes groups of infected animals (E-coli) or controls (saline). Error bars indicate ​± ​one SEM. Each point represents an average of values obtained from animals within one litter of the same condition. Shaded squares are carrageenan treated animals and filled circles are saline controls. There is a significant learned aversion to painful stimuli in adults exposed to inflammatory injury with carrageenan (F (1,17) ​= ​5.631, p ​= ​0.030) and in adults with combined exposure to E-coli and carrageenan early in life (F (1,17) ​= ​5.728, p ​= ​0.028).

The 3-way ANOVAs (Carrageenan by E-coli by Sex) showed no sex differences so male and female data were combined (Fig. 5; Table 1). Subsequent 2-way ANOVAs showed that early inflammatory injury and E-coli infection did not significantly alter pain affect on PN8 (all p values ​> ​0.547) or PN15 (all p values ​> ​0.136), as measured by learned aversion in CPA testing. In adulthood, animals that experienced early inflammatory injury with carrageenan spent less time in the test chamber associated with the painful stimulus than controls, demonstrating an increased aversion to painful stimulation (F (1,17) ​= ​5.631, p ​= ​0.030; Cohen’s d ​= ​0.785). E-coli infection, which alone had no effect (F (1,17) ​= ​0.325, p ​= ​0.576), when combined with carrageenan injury significantly increased aversion to painful stimulation in adulthood (F (1,17) ​= ​5.728, p ​= ​0.029; Fig. 5). Posthoc analysis showed that carrageenan injured animals show more aversion than the saline treated controls when infected with E-coli as infants (p-value ​= ​0.013; Cohen’s d ​= ​2.690) but no significant aversion in the absence of early infection (p-value ​= ​0.987). Thus E-coli infection and early carrageenan injury combined to increase pain aversiveness in adulthood to a greater extent than either insult alone.

## 4 Discussion

Characterizing the newborn response to painful stimulation is a necessary first step in understanding how pain is perceived and processed in this vulnerable patient population. Clinical and laboratory studies have shown alterations in HPA axis (Brummelte et al., 2015; Chen et al., 2016; Gover et al., 2013; Grunau et al., 2013; Holsti et al., 2007; Provenzi et al., 2016), pain response, and behavior associated with pain and stress experienced early in life (Anand, 1998; Andrews and Fitzgerald, 1994; Bouza, 2009; Fitzgerald, 2005; Grunau, 2002; Johnston et al., 2002; Oberlander et al., 2000; Ranger and Grunau, 2014; Taddio et al., 1995, 1997; Verriotis et al., 2016; Whitfield and Grunau, 2000). Although the effects of early inflammatory tissue injury on nociception in newborns has been extensively studied (Andrews and Fitzgerald, 1994; Fitzgerald, 2005; Koch and Fitzgerald, 2013), its effects on the affective component of the pain response are not as well described. In addition data on how external variables, including comorbidities of prematurity, interact to collectively influence pain response remains limited. As newborns in intensive care settings are commonly and concurrently exposed to both early painful experiences and early infection, it is critical to understand the combined effects of these experiences on behavior and development. To address these gaps in knowledge, we exposed rat pups to injury with low-dose carrageenan and E-coli infection and found that early painful injury and early infection have short-term and long-term effects on nociception and long-term effects on the affective components of the pain response.

Newborn E-coli infection and neonatal pain induced by carrageenan both independently increased nociceptive scores following hind paw injury with formalin on PN8, with effects persisting into adulthood in the carrageen group. When experienced concurrently, these early exposures to pain and infection produced hyperalgesia in formalin testing on PN8 and contributed to the long-term development of aversion to pain. Although early carrageenan injury alone also significantly increased pain aversion in adults, this effect was largely driven by its interaction with E-coli. The main effect size of carrageenan alone was small as compared to the reported values of other effects. Thus the carrageenan effects stem from its interactions with E-coli. In this light, there was a strong effect of E-coli combined with carrageenan for males only at PN8 (Table 2), which is consistent with an increased vulnerability of premature human male babies. These results demonstrate age-dependent interactions between newborn injury and infection and long-term pain-related outcomes. To our knowledge this is the first report of the combined effects of neonatal inflammatory injury and systemic E-coli infection on pain response throughout development.

### 4.1 Comparison to the human experience

In this study, every effort was made to ensure that both the timing of infection (PN2) and inflammatory injury (PN3) closely paralleled the clinical experience of babies in the NICU. All experimental exposures occurred within 72 ​h of birth because brain development at this time point in rat pups is most similar to that of premature human infants at the limits of viability [e.g. (Semple et al., 2013; Workman et al., 2013)]. E-coli inoculation was specifically used in lieu of other methods of immune system activation, including LPS, as E-coli commonly causes early onset sepsis in preterm infants (Schrag et al., 2016; Schrag and Stoll, 2006). Lastly, carrageenan injection of the paw was used to mimic inflammatory tissue injury seen after heel sticks, venipunctures, and other skin breaking procedures that are frequently performed during newborn intensive care. This design allowed us to directly assess the independent and combined effects of separate pro-inflammatory processes on both the nociceptive and affective components of the behavioral response to pain throughout development. It also allowed us to compare localized painful inflammatory injury with carrageenan to systemic inflammatory processes with E-coli.

### 4.2 Parallels to the prior literature

Long term effects on basal pain sensitivity following early painful injury are mixed (Anand et al., 1999; Lidow et al., 2001; Low and Fitzgerald, 2012; Ren et al., 2004b; Ruda et al., 2000). Lidow and colleagues found that injection of low-dose carrageenan into the hindpaw of pups on PN0– PN5 resulted in basal thermal hypoalgesia in the absence of ongoing inflammatory insult (Lidow et al., 2001; Ren et al., 2004b). In our experiments, however, carrageenan exposure on PN3 had no significant long-term effects on baseline thermal heat latencies. Although similar phenotypic changes, including hindpaw swelling and erythema, were observed after carrageenan injection [52–55], there were no differences in heat latencies in exposed animals at any time point in our study, a finding consistent with other studies where repetitive injury to the hindpaw early in life had no effects on hot plate latencies in adulthood (Anand et al., 1999). Similarly, animals subjected to hindpaw injection with inflammatory CFA (Ruda et al., 2000) or repetitive plantar incisions in the neonatal period (Low and Fitzgerald, 2012) had no significant alterations in baseline mechanical (Low and Fitzgerald, 2012) or thermal heat latencies as adults (Low and Fitzgerald, 2012; Ruda et al., 2000). This collectively suggests that early inflammatory injury does not always alter baseline sensitivity to thermal stimuli. The reasons for the variable effects in the thermal test are unclear. Lack of effect in our study may be due to differences in study design including choice and dose of inflammatory agent used, duration and severity of the initial inflammatory injury (Laprairie and Murphy, 2007), and choice of behavioral testing performed. Another possible explanation is that our animals were bred in our home colony whereas animals used in others’ work were shipped pregnant. Given that prenatal stress has been demonstrated to enhance pain mechanisms (Knaepen et al., 2013; Sternberg and Ridgway, 2003), it is possible that the gestational stress of long-range transport adds to the developmental plasticity of pain in pups.

Exposure to noxious stimulation in the newborn period has been associated with long-term hyperalgesia after repeat injury (Ren et al., 2004a; Ruda et al., 2000; Taddio et al., 1995, 1997). Our study similarly showed increased pain scores after formalin reinjury on PN8 and in adulthood in animals exposed to carrageenan early in life. Alterations in the behavioral response to formalin reinjury in adulthood have been most consistently seen in studies where aggressive early injury caused a significant or prolonged inflammatory state (Anand et al., 1999; Ruda et al., 2000). Thus, it seems that both the severity and duration of injury play a key role in influencing pain behaviors. The increased pain scores we observed in carrageenan exposed groups on PN8 may have been due to the presence of residual inflammation. Following resolution of the initial inflammatory process, there were no longer any differences in pain behavior observed between groups as noted in formalin testing on PN15. The observed effects of early injury on adult pain behavior, therefore, could not be a direct result of ongoing inflammation and may instead be due to mechanistic changes that occur over time secondary to that initial inflammatory tissue injury. Although additional studies are necessary to investigate the mechanisms underlying these longstanding changes in behavior, our findings clearly demonstrate that painful inflammatory tissue injury experienced in early life is associated with a heightened pain response both short-term and long-term. This led us to wonder if simultaneous exposure to multiple pro-inflammatory processes early in life might synergistically influence this observed pain response.

In addition to skin breaking procedures, systemic infections are a recognized complication of being born too soon (Baley and Leonard, 2013; Carbajal et al., 2008; Schrag et al., 2016; Schrag and Stoll, 2006). Several clinical studies have established associations between neonatal pain and long-term adverse neurodevelopmental outcomes (Bhutta and Anand, 2002; Bouza, 2009; Chau et al., 2013; Duerden et al., 2018; Lowery et al., 2007; Morag et al., 2017; Valeri et al., 2015; Walker, 2013) and early immune system activation in the setting of infection has similarly been associated with long-term changes in adult behavior (Bilbo et al., 2005b; Bilbo and Schwarz, 2012). Infection and tissue injury are both frequently encountered during routine newborn intensive care but little is known about their collective effects on pain response during different stages of development. Pain scores were highest in pups with simultaneous exposure to both early carrageenan injury and E-coli, suggesting that pro-inflammatory processes can collectively alter the nociceptive component of the behavioral pain response short-term.

### 4.3 Importance of pain affect

The affective component of pain Is equally important to the nociceptive component, yet seldom studied. “Suffering”, classically associated with pain, is largely due to how painful experiences make us feel. This goes beyond spinal level reflexes and nociception to involve higher order processing in the brain. In our experiments, we used a classical conditioning paradigm to study learned aversion to painful stimuli. Aversion to pain developed over time, with animals at PN8 still showing a relative preference for the chamber associated with a painful stimulus, as might be expected at this age. This paradoxical finding has been previously demonstrated in animal models of abusive caregivers (Sullivan et al., 2000) where altricial species classically develop a preference for stimuli associated with their caregivers, even if these stimuli are noxious (Moriceau and Sullivan, 2005; Roth et al., 2013; Sullivan et al., 2000). By PN15, when altricial species have increased mobility and decreased dependence on their caregiver (Moriceau and Sullivan, 2005), there was a trend towards development of aversion to the painful stimulus in animals exposed to both carrageenan and E-coli early in life. By adulthood, this aversion to the painful stimulus was clearly significant, and most prominent in those pups exposed to both inflammatory injury and systemic infection early in life. These findings demonstrate the synergistic and longstanding effects of pro-inflammatory processes on the affective component of the pain response. It is important to note that the strain of E-coli used in our experiments produced only mild and self-limited illness whereas systemic infection with E-coli or other bacteria in pre-term babies often produces severe and life-threatening disease. The degree of inflammation encountered clinically is higher than what we could reasonably model in our experiments in order to ensure survivability of pups to adulthood. Given our current findings, we would expect that inoculation with a more virulent strain would result in a greater degree of illness and inflammation, leading to more heighted aversion to pain in surviving animals.

### 4.4 Possible mechanisms

Determining changes in neural processing due to the combination of pain and infection was beyond the goals of this study but there are several possible mechanisms that can be hypothesized. Among the possible neural changes that could account for our findings, the PAG, spinal cord, and cerebral cortex, have been shown to be altered by pain and infection [in humans: (Duerden et al., 2018; Ranger et al., 2013; Vinall et al., 2014); in animal models (Anand et al., 1999; Andrews and Fitzgerald, 1994; Ruda et al., 2000; Yan and Kentner, 2017; Zouikr et al., 2014a, 2014b)]. Moreover, many of these same structures show augmented immune responses in adulthood following infant infection (Campbell et al., 2015; Wang et al., 2011; Zouikr et al., 2014a, 2014b, 2015). It will be important to determine if infant infection concurrent with pain enhances (or dampens) changes in the neural circuit activity or in the activation of immune responses in these same circuits.

Pain affect is regulated by overlapping and independent brain circuitry. One such circuit is the spinal cord dorsal horn to the parabrachial nucleus to the central and basolateral amygdala, with output to the PAG and communication with the anterior cingulate cortex to modulate pain (Minami, 2009; Neugebauer, 2015). Early injury alters this circuit; for example, enkephalin mRNA and protein are elevated in the central nucleus of the amygdala accompanied by dampened stress and anxiety responses in behavioral assays (Victoria et al., 2013) whereas early infection also increases pain sensitivity but not anxiety or depressive-like behaviors (Yan and Kentner, 2017). Brain circuitry engaged in pain processing in infants is very similar to that in adults with the important exception of the amygdala (Goksan et al., 2015). In animal models, the amygdala is quiescent in early life, resulting in paradoxical learned preferences for cues associated with noxious stimulation (Sullivan et al., 2000). The amygdala can be activated by systemic administration of corticosterone resulting in the expected, and adult-like learned aversions to cues associated with pain (Moriceau et al., 2004). This premature activation of the amygdala then remains abnormally responsive to aversive and social cues in adults [reviewed in (Santiago et al., 2017)]. We can speculate that the combination of E-coli and carrageenan might have elevated corticosterone sufficiently to result in premature activation of the amygdala, which is then permanent, and which in turn had long-term consequences for the enhanced aversion to pain in the adults.

## 5 Limitations

There are several limitations to this work. First, we used a single injury to the paw at a single age. In the NICU, skin breaking events occur multiple times during a day and over the course of days to months. Whether a more intense stimulus such as repeated paw stabs (Anand et al., 1999) would have different effects when combined with infection remains unknown. Likewise, our model used a relatively mild and time limited infection to ensure survival of the pups. Systemic E-coli infections in newborns are longer lasting, more severe, often associated with increased morbidity and mortality, and always treated with antibiotics. In addition, it would have been helpful to continue measuring levels of corticosterone, ACTH, and interleukin 1β over time in order to better estimate the exact duration of the E-coli infection and associated inflammatory response induced by our model.

We tested only baseline thermal nociception for the acute pain test and there were no effects of early treatment. Other tests, mechanical, cold, visceral might be more sensitive to early injury and infection. Additionally, we used the CPA test as a measure of pain affect. One potential limitation to this test is that it involves learning. Our test of necessity was a single trial. If our infant manipulations altered learning capabilities, that might have reduced the effectiveness of the CPA test. Other measures, such as a grimace test, could provide additional assessments of the negative affect associated with pain.

Finally, we did not identify the mechanisms underlying the enduring behavioral changes described above. There are multiple possible ways in which the combination of pain and infection could alter adult behaviors that are not influenced by either alone. These could include different circuits, different activation of similar circuits, and changes in how later immune and inflammatory processes are engaged by adult pain.

## 6 Conclusions and future directions

Our study demonstrates a robust, reproducible, and long-standing change in pain behavior after neonatal exposure to infection and painful stimulation that has not been previously described. Whereas exposure to carrageenan and systemic infection early in life did not alter acute sensitivity to detection of painful stimuli in pups, each resulted in an increased behavioral response to evoked pain in infancy and development of a long-term aversion to pain in adults. Our findings suggest a compounding effect of pro-inflammatory processes on pain behaviors at developmental stages in pups that correlate with postmenstrual ages encountered in the NICU. Additionally, affected animals go on to have increased aversion to painful stimuli in adulthood, highlighting how early life experiences can alter adult behavior long-term. These findings are particularly important in the context of neonatal intensive care, where painful stimulation in preterm babies is common and often occurring concurrently with systemic infection.

The next logical step is to identify the mechanisms underlying these long-term changes in behavior. With the behavioral effects well-characterized, follow-up studies can focus on uncovering those mechanisms. Possible studies include defining long lasting changes in CORT release, characterizing altered activity in structures implicated in the long-term effects of either infection or early pain (including mPFC, hippocampus, amygdala), or assessing how exposure to early pain/early infection changes neuronal firing under both resting conditions and following injury. Use of alternative models of infection (LPS, Streptococcus, viral infection etc.) would provide clues as to how generalizable behavioral effects observed following other modes of immune system activation are to our findings with E-coli. Finally, inclusion of other clinical variables, such as treatments for pain, infection or both, into this model would be important to mimic the clinical setting.

## Declaration of competing interest

The authors have no conflicts of interest to disclose.
