# Supplementary figures and images for: Local injury and systemic infection in infants alter later nociception and pain affect during early life and adulthood
Source: Brain Behav Immun Health. 2020 Nov 10;9:100175. doi: 10.1016/j.bbih.2020.100175 (PMC8474633; doi:10.1016/j.bbih.2020.100175)

# Infection

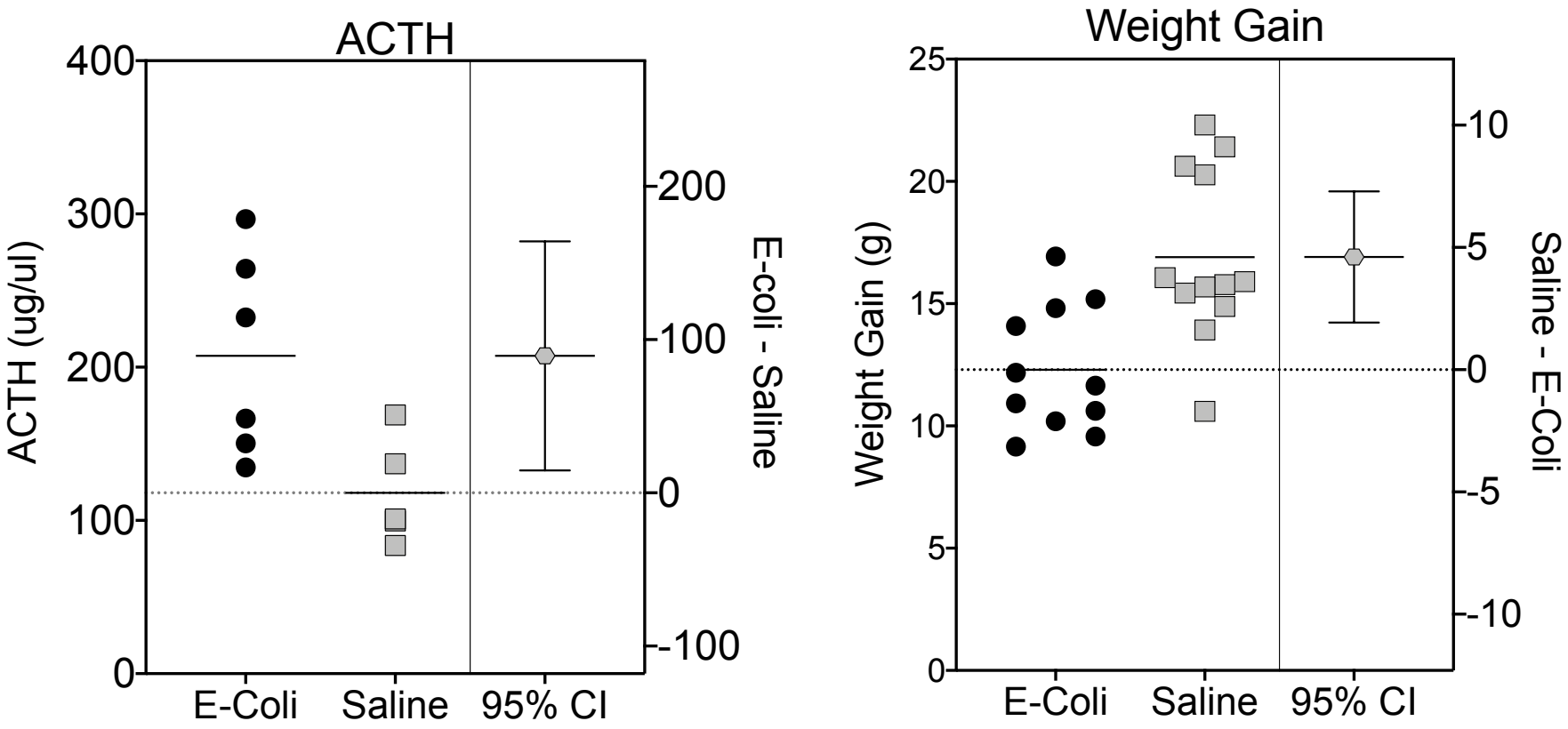

Supplement: Multimedia component 1 [file mmc1.pdf]

# PN8 Formalin Test

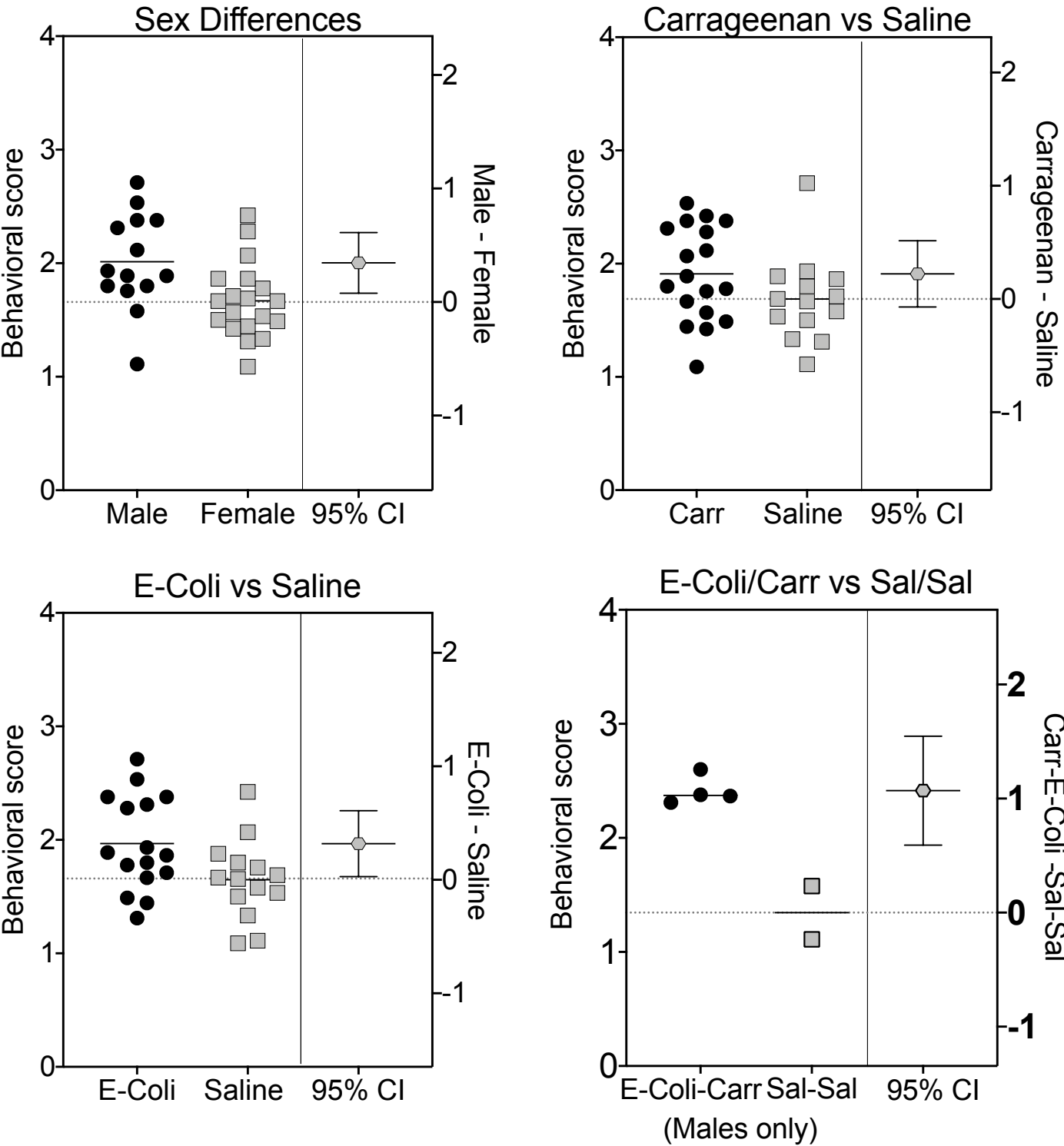

Supplement: Multimedia component 2 [file mmc2.pdf]

## Adult Formalin

Sex Differences

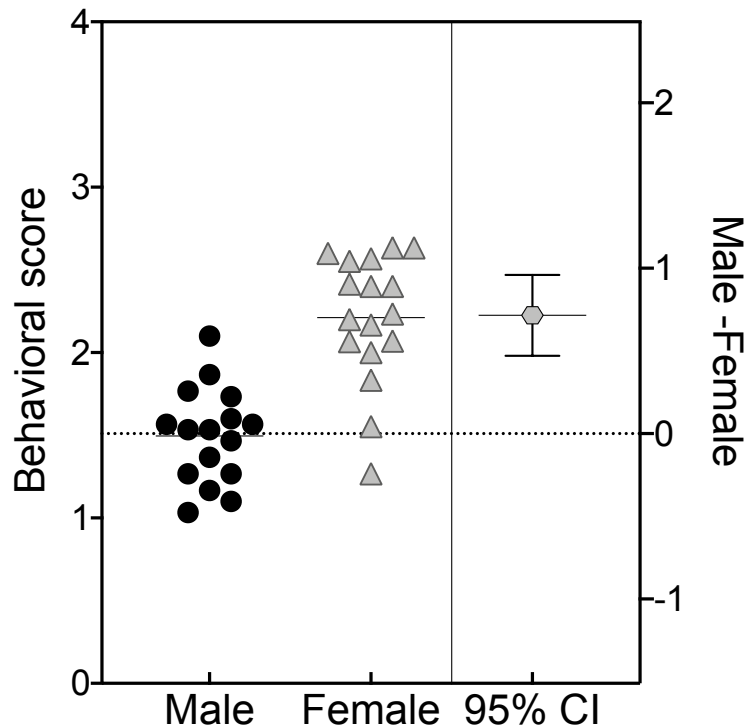

Carrageenan vs Saline

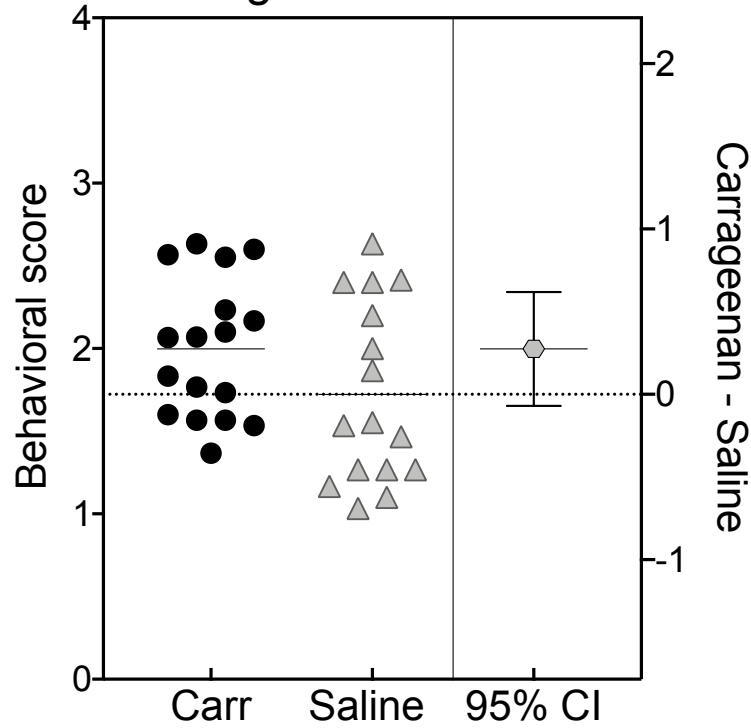

Supplement: Multimedia component 3 [file mmc3.pdf]

# Adult CPA

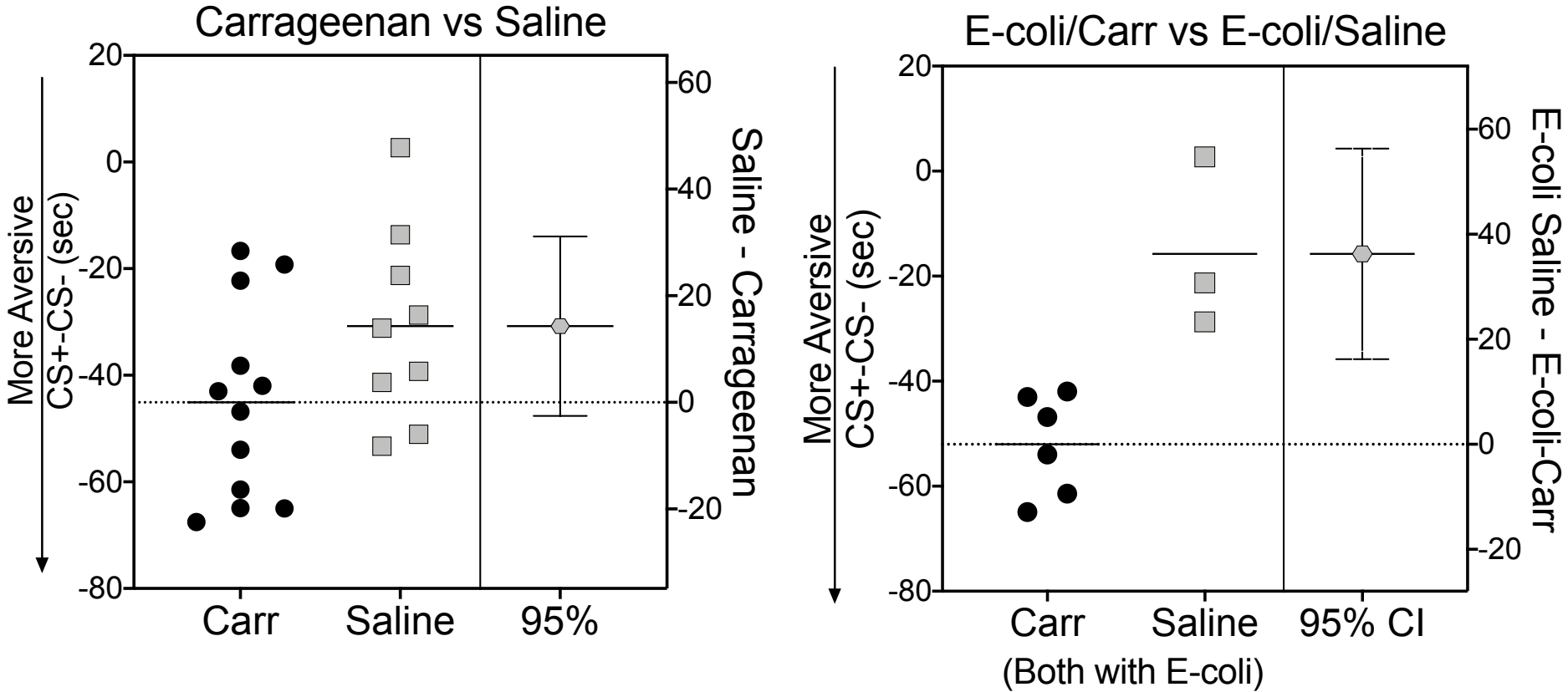

Supplement: Multimedia component 4 [file mmc4.pdf]

**PN 8 Paw Difference**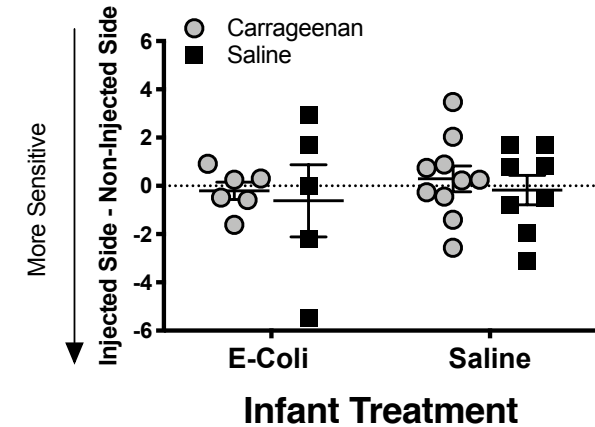**PN 15 Paw Difference**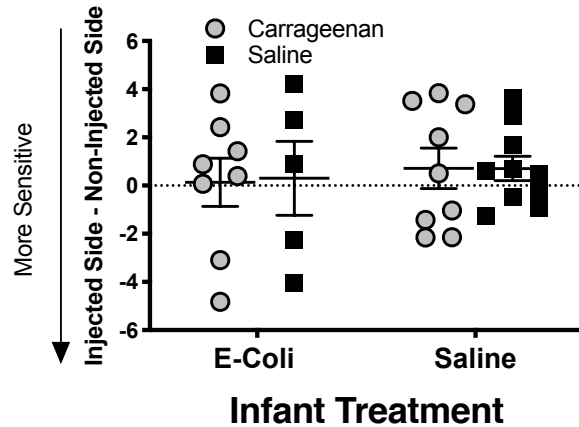**Adult Paw Difference**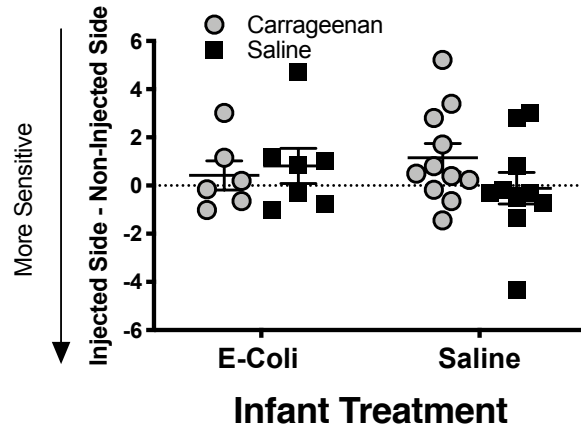

Supplement: Multimedia component 6 [file mmc6.pdf]
