# Supplementary material for: Local injury and systemic infection in infants alter later nociception and pain affect during early life and adulthood
Source: Brain Behav Immun Health. 2020 Nov 10;9:100175. doi: 10.1016/j.bbih.2020.100175 (PMC8474633; doi:10.1016/j.bbih.2020.100175)
Supplement: Multimedia component 5 [file mmc5.docx]

**SUPPLEMENTAL FIGURE LEGENDS**

**Supplemental Figure 1:** The left panel shows the estimation plot for serum concentration of ACTH and the right panel shows the estimation plot of percent? weight gained in the 24h after E-coli injection (difference in weight between PN2 and PN3). The individual data points are shown to the left of the vertical solid line in each panel and scaled to the left Y-axis. The mean difference score (E-coli minus Saline for ACTH or Saline minus E-coli for Weight Gain) between the two groups is shown to the right of the vertical solid line in each panel and scaled to the right Y-axis with the 95% confidence interval (CI) indicated by the ends of the horizontal error bars. The horizontal dashed line corresponds to? The left Y-axis shows concentration of ACTH in the left panel, and percent weight gain in the right panel. The X-axis denotes groups of infected animals (E-coli) and controls (saline). As can be seen in both panels, the mean of the comparison group (E-coli on the left; Saline on the right) is outside that 95% CI providing confidence that the two groups differed. This provides evidence for successful infection with E-coli.

**Supplemental Figure 2:** These 4 panels depict the estimation plots for the formalin test at PN8. Specifically, estimation plots are shown for effects of sex, carrageenan exposure, E-coli infection, and combined exposure to both E-coli and carrageenan. Details of the graphs are as in Supplemental Figure 1. For all panels behavioral (pain) score is shown on the left Y-axis. The X-axis for each panel denotes the groups being compared. For three of the effects (Sex Differences, E-coli, and the interaction of E-coli with Carrageenan in males), the effects were robust with the 95% CI of the difference score not overlapping between groups. This is consistent with the robust Cohen’s d effect size (Table 2) and the statistical tests. For the Carrageenan vs Saline comparison, there was overlap of the 95% CI suggesting a less robust effect despite significance in the overall ANOVA. This is consistent with the smaller Cohen’s d effect size and non-significant two-group t-test (Table 2).

**Supplemental Figure 3:** These 2 panels depict the estimation plots for the formalin test in adults. Specifically, estimation plots are shown for effects of sex and carrageenan exposure. Details of the graphs are as in Supplemental Figure 1. For both panels behavioral (pain) score is shown on the left Y-axis. The X-axis for each panel denotes the groups being compared. Sex differences in the left panel were quite robust with no overlap of the 95% CI. In contrast, as at PN8, the Carrageenan vs Saline comparison showed overlap suggesting a less robust difference. This is consistent with the smaller Cohen’s d effect size and non-significant two-group t-test (Table 2).

**Supplemental Figure 4:** These 2 panels depict the estimation plots for the Conditioned Place Aversion Test (CPA) in adults. Specifically, estimation plots are shown for effects of carrageenan exposure and combined exposure to E-coli infection and carrageenan. Details of the graphs are as in Supplemental Figure 1. For both panels the left Y-axis denotes the relative amount of time spent in the testing chamber paired with the painful stimulus. On this scale, positive numbers indicate a relative preference for the chamber associated with painful experience (more time spent in the chamber) whereas negative numbers indicate a relative aversion (less time spent in the chamber). The X-axis denotes groups being compared. Although there was a significant Carrageenan/Saline difference in the ANOVA, the data suggest it was driven by the interaction with E-coli (Figure 4). This conclusion was strengthened by the relatively smaller Cohen’s d effect size (Table 2) and a non-significant t-test between the two groups. On the right we show the interaction effect in which the combination of E-coli with Carrageenan produced a greater aversion to the formalin-associated test cues than did E-coli alone as evidenced by the lack of overlap with the 95% CI.

**Supplemental Figure 5:** These panels show the difference in baseline thermal thresholds of the carrageenan injected (left) paw and the uninjected (right) paw at three developmental time points (PN8, PN15, and in adults). The developmental time point studied is shown at the top of each panel. The X-axis denotes groups of infected animals (E-coli) and controls (saline). The Y-axis represents difference in withdrawal latencies in seconds? There were no differences in heat latencies at any age, with or without prior E-coli infection. These data support those shown in Supplemental Table 1.
