# Supplementary material for: Local injury and systemic infection in infants alter later nociception and pain affect during early life and adulthood
Source: Brain Behav Immun Health. 2020 Nov 10;9:100175. doi: 10.1016/j.bbih.2020.100175 (PMC8474633; doi:10.1016/j.bbih.2020.100175)
Supplement: Multimedia component 7 [file mmc7.docx]

**Supplementary Table 1.**

Differences in thermal test between injected and the contralateral paw.

**Age/Tx Mean Left Mean Right Mean Diff Mean Ratio**

| PN 8 Saline |  |  |  |  |
| --- | --- | --- | --- | --- |
| Carrageenan | 14.44 | 14.15 | 0.29 | 1.04 |
| Saline | 12.80 | 12.97 | -0.17 | 1.00 |
|  |  |  |  |  |
| PN8 E-coli |  |  |  |  |
| Carrageenan | 13.16 | 13.37 | -0.21 | 0.99 |
| Saline | 12.79 | 13.13 | -0.62 | 0.97 |
|  |  |  |  |  |
| PN 15 Saline |  |  |  |  |
| Carrageenan | 12.42 | 11.71 | 0.72 | 1.08 |
| Saline | 12.99 | 12.28 | 0.71 | 1.07 |
|  |  |  |  |  |
| PN 15 E-coli |  |  |  |  |
| Carrageenan | 13.43 | 13.29 | 0.14 | 1.02 |
| Saline | 12.31 | 11.94 | 0.30 | 1.01 |
|  |  |  |  |  |
| Adult Saline |  |  |  |  |
| Carrageenan | 9.95 | 8.79 | 1.16 | 1.13 |
| Saline | 8.13 | 8.24 | -0.11 | 1.01 |
|  |  |  |  |  |
| Adult E-coli |  |  |  |  |
| Carrageenan | 9.85 | 9.42 | 0.42 | 1.05 |
| Saline | 8.66 | 7.85 | 0.82 | 1.07 |

**Note**- The left paw was injected with carrageenan or saline on PN3. The difference means are the left paw – the right paw. Likewise the ratio means are the left paw/right paw. The entries are the withdrawal latency in seconds at each age and E-coli/saline treatment (PN2). There are no significant differences.
